# Situational Awareness and Proactive Engagement Predict Higher Time in Range in Adolescents and Young Adults Using Hybrid Closed-Loop

**DOI:** 10.1155/2023/1888738

**Published:** 2023-05-18

**Authors:** Laurel H. Messer, Paul F. Cook, Stephen Voida, Casey Fiesler, Emily Fivekiller, Chinmay Agrawal, Tian Xu, Gregory P. Forlenza, Sriram Sankaranarayanan

**Affiliations:** 1 Barbara Davis Center for Diabetes, University of Colorado Anschutz Medical Campus, Aurora, CO, USA; 2 College of Nursing, University of Colorado Anschutz Medical Campus, Aurora, CO, USA; 3 Department of Information Science, University of Colorado Boulder, Boulder, CO, USA; 4 Department of Computer Science, University of Colorado Boulder, Boulder, CO, USA

## Abstract

**Background.:**

Adolescents and young adults with type 1 diabetes have high HbA1c levels and often struggle with self-management behaviors and attention to diabetes care. Hybrid closed-loop systems (HCL) like the t:slim X2 with Control-IQ technology (Control-IQ) can help improve glycemic control. The purpose of this study is to assess adolescents’ situational awareness of their glucose control and engagement with the Control-IQ system to determine significant factors in daily glycemic control.

**Methods.:**

Adolescents (15–25 years) using Control-IQ participated in a 2-week prospective study, gathering detailed information about Control-IQ system engagements (boluses, alerts, and so on) and asking the participants’ age and gender about their awareness of glucose levels 2–3 times/day without checking. Mixed models assessed which behaviors and awareness items correlated with time in range (TIR, 70–180 mg/dl, 3.9–10.0 mmol/L).

**Results.:**

Eighteen adolescents/young adults (mean age 18 ± 1.86 years and 86% White non-Hispanic) completed the study. Situational awareness of glucose levels did not correlate with time since the last glucose check (*p* = 0.8). In multivariable modeling, lower TIR was predicted on days when adolescents underestimated their glucose levels (*r* = −0.22), received more CGM alerts (*r* = −0.31), and had more pump engagements (*r* = −0.27). A higher TIR was predicted when adolescents responded to CGM alerts (*r* = 0.20) and entered carbohydrates into the bolus calculator (*r* = 0.49).

**Conclusion.:**

Situational awareness is an independent predictor of TIR and may provide insight into patterns of attention and focus that could positively influence glycemic outcomes in adolescents. Proactive engagements predict better TIR, whereas reactive engagement predicted lower TIR. Future interventions could be designed to train users to develop awareness and expertise in effective diabetes self-management.

## 1. Introduction

Adolescents and emergent adults (ages 15–25 years) with T1D have the highest average HbA1c levels of any age group with diabetes, peaking at 9.3%, well above goal of 7.0% for most people with diabetes [1]. Diabetes technologies such as hybrid closed-loop systems (HCL) can improve glycemic control in children, adolescents, and adults with diabetes [2–5]. HCL systems partially automate insulin delivery with algorithms that use sensor glucose input to administer insulin doses aimed at keeping glucose levels in target range. The Tandem t:slim X2 with Control-IQ technology (referred to here as “Control-IQ”) is one of these HCL systems. The Control-IQ system consists of an insulin pump that implements the Control-IQ algorithm combined with a Dexcom G6 continuous glucose monitor (CGM) [6]. Persons with diabetes who use the Control-IQ system wear the system continuously and direct the pump to deliver insulin boluses for meals and hyperglycemia as needed.

While the Control-IQ system improves glycemic control in adolescents, user behavior and engagement remain important to achieving optimal glycemia [7, 8]. Diabetes self-management behaviors are particularly difficult for adolescents and young adults due to a variety of developmental, cognitive, and psychological factors unique to the age group [9–11]. Engagement with HCL systems like Control-IQ (e.g. giving insulin boluses, monitoring glucose levels, and so on) is one subset of self-management, together with other behaviors like food selection or timing, and physical activity. We have previously shown how adolescents and young adults have high interpersonal and intrapersonal variability in their diabetes self-management behaviors, and how a variety of biopsychosocial daily factors correlate with these fluctuations [12]. Therefore, more research about their engagement with their diabetes care is warranted.

Because adolescents face many competing challenges for attention, we examined how “situationally aware” adolescents and young adults were in relation to their diabetes care throughout the day. Situational awareness is defined as a combination of (a) knowing numerous pieces of data, (b) having a deep understanding of context, and (c) being able to project future states in reference to present goals [13]. Situational awareness is associated with expertise, and the related cognitive processes often bypass conscious awareness [14]. In the context of diabetes, situational awareness refers to “strategic” awareness of current health states, an understanding of what they mean, and the ability to execute self-management behaviors that affect them. Among adults with diabetes, greater skill in recognizing glucose problems was linked to better glucose control in a way that declarative knowledge about diabetes was not [15]. Situational awareness involves automatic perception and attention processes that we have characterized as belonging to the “Intuitive mind,” which can be differentiated from factual knowledge and intentions at the more conscious “narrative mind” level [16].

The purpose of this study, therefore, was to assess adolescents’ engagement with the Control-IQ system, and their situational awareness of glucose levels throughout the day, and to evaluate these variables’ effects on glycemic control. Identifying patterns of awareness, engagement, and glycemia is a first step to understand how adolescents and young adults can more effectively manage their diabetes using the Control-IQ system.

## 2. Methods

We conducted a prospective, 2-week study involving adolescents and young adults recruited from the Barbara Davis Center to collect data related to diabetes engagement, situational awareness, and glucose control. The Colorado Multiple Institutional Review Board approved this research. Participants were ages 15–25 years old inclusive, had a diagnosis of type 1 diabetes, and used the Control-IQ system to manage their diabetes. Our intention was to recruit individuals with diverse HbA1c levels at baseline, so potential participants were prescreened for this and use of Control-IQ. We chose Control-IQ as the HCL system of interest because it was the most widely used HCL in our clinic at the time of the study. Additionally, participants had to be using a commercial iOS (Apple) iPhone device with the HealthKit application and be willing to wear a compatible smartwatch for the duration of the study.

### 2.1. Procedures.

Participants were enrolled in this study for 2-weeks and wore their Control-IQ system continuously. Although during routine use, Control-IQ users can check glucose levels on the insulin pump itself or a separate CGM app on a phone, we asked participants to only check glucose levels on their insulin pump so we could collect data about these interactions from the pump itself to better quantify user engagement. Throughout the study period, participants were sent 2–3 quasi-randomly timed “situational awareness” surveys that asked about their current awareness of glucose levels and predictions for future glucose levels.

### 2.2. Data Collection

#### 2.2.1. Situational Awareness Questionnaire.

Situational awareness can be difficult to measure, but studies have shown that “awareness-in-the-moment” measures are more correlated with performance than a participant’s subjective rating of how situationally aware they were after the fact [17]. We therefore assessed situational awareness with a 4-item survey delivered at random times and asked participants: (1) when did you last view your CGM glucose value? (In the past 15 minutes, past hour, past 2 hours, past 3 hours, and longer than 3 hours); (2) without looking, what is your glucose now? (<70 mg/dl, 71–120 mg/dl, 121–180 mg/dl, 181–250 mg/dl, and >250 mg/dl); and (3) without looking, what direction is it trending? (going up, going down, and staying stable); and (4) after looking at your CGM, what do you think your glucose level will be 1 hour from now? (<70 mg/dl, 71–120 mg/dl, 121–180 mg/dl, 181–250 mg/dl, and >250 mg/dl). Items 1 and 2 correspond to knowledge of data, item 3 reflects contextual knowledge, and item 4 requires prediction, which are the three major components of situational awareness. We expected that all of these components would predict glycemic control.

#### 2.2.2. Engagement with CGM.

Engagement behaviors were quantified from Control-IQ downloads, including the daily number of interactions with the Control-IQ system (including checking glucose levels, responding to alerts, giving meal boluses, and pump maintenance), the number of CGM alerts, the percent of CGM alerts acknowledged by the user, the number of boluses given, the number of grams of carbohydrate entered into the pump each day, and the number and percent of boluses that included a carbohydrate entry. Each of these system device functions can be part of a participant’s diabetes self-management approach, but at the outset we did not have any clear expectation about which of them might be most strongly correlated with glycemic control.

#### 2.2.3. Glucose Outcome.

Daily glucose data were collected from the CGM, with time-in-range (TIR, 70–180 mg/dl, 3.9 to 10.0 mmol/L) as the primary outcome variable.

### 2.3. Analysis.

Participant demographics and missing data patterns were examined descriptively, with complete data available on the outcome variable (TIR) and no systematic pattern of missingness for predictors. We used a two-stage analysis to examine the effects of Control-IQ engagement and situational awareness on TIR. First, we analyzed individual participant characteristics as predictors of TIR, including within-person average scores on the situational awareness and Control-IQ engagement variables over the full 2 weeks of data collection, as well as stable demographic characteristics. With 14 participants, we had 80% power to detect only moderate to large effects of *r* = 0.48 or greater at *α* = 0.05, so Type II error is a potential concern in these analyses.

Second, we analyzed day-to-day variation in TIR within participants, which was substantial (ICC [2] = 0.497 for TIR, meaning that about 50% of all variability in TIR was within-persons). In the context of high day-to-day variability, it makes sense to also examine predictors of TIR on a daily basis; in contrast to the person-level effects, these tests examine more transient relationships between a person’s situational awareness and their TIR at a specific point in time. Variables that predict within-person changes in TIR could be valid targets for tailored interventions that attempt to improve diabetes self-management at the specific moment when an intervention would be most beneficial. To test within-person relationships, we used linear mixed models in SPSS v28.0, with restricted maximum likelihood estimation. Control-IQ engagement and situational awareness variables were each tested in separate models, and significant predictors were then combined in a single model to address multicollinearity. We tested fixed effects for all predictors and used an AR (1) data structure to account for the autoregression noted in the TIR values, *r* = 0.69 for the correlation between each day’s TIR value and the ones before or after it. Because there was a slight linear trend in TIR (showing improved daily glucose control from start to end of the 2-week monitoring period), we also included study day as a covariate in all models. Finally, to increase confidence in causal associations, we also tested time-lagged models in which the self-management variable on one day was used to predict the outcome variable on the following day. These tests add to the utility of the within-person tests by examining the same effects after a slight time lag; significant effects in this context suggest a prospective (and therefore potentially causal) relationship between the situational awareness variable and TIR. Power for the within-person tests was slightly higher—80% to detect effects as small as *r* = 0.42 at *α* = 0.05, based on an average of 11.5 data points each from 14 participants with an intraclass correlation of 0.71, resulting in an effective sample size of 19 for the multilevel analyses [18].

## 3. Results

We contacted 19 individuals for participation. One declined, 2 did not respond, 1 did not show up for appointment, and 1 did not meet eligibility criteria. A total of 14 adolescents/young adults were enrolled in the study (64% female and 86% White non-Hispanic, Table 1). Three participants had been using Control-IQ less than one year, 8 for 1–2 years, and 3 for 2–3 years.

### 3.1. Predictors of TIR Differences between Participants.

The participants’ age, gender, duration of diabetes, years using Control-IQ, insurance status, and parent’s education level did not predict TIR. The participant’s baseline HbA1c level did predict TIR (*p* < 0.001).

Situational awareness questionnaire responses and diabetes behaviors were averaged for each individual and tested for correlation with average TIR. Situational awareness responses did not correlate with the amount of time since the individual last checked their glucose level (*r* = 0.08, *p* = 0.8). The percent of time an individual underestimated their glucose level negatively correlated to TIR (*r* = −0.73, *p* = 0.02, Table 2). For diabetes behaviors, the percent of CGM alerts that were acknowledged by the user strongly correlated to TIR (*r* = 0.76, *p* < 0.01), and the mean number of CGM alerts negatively correlated to TIR (*r* = −0.43, *p* = 0.01). Both the total grams of carbohydrates entered into the pump and the mean number of boluses with carbohydrate entries moderately correlated with TIR.

**Figure 1:**
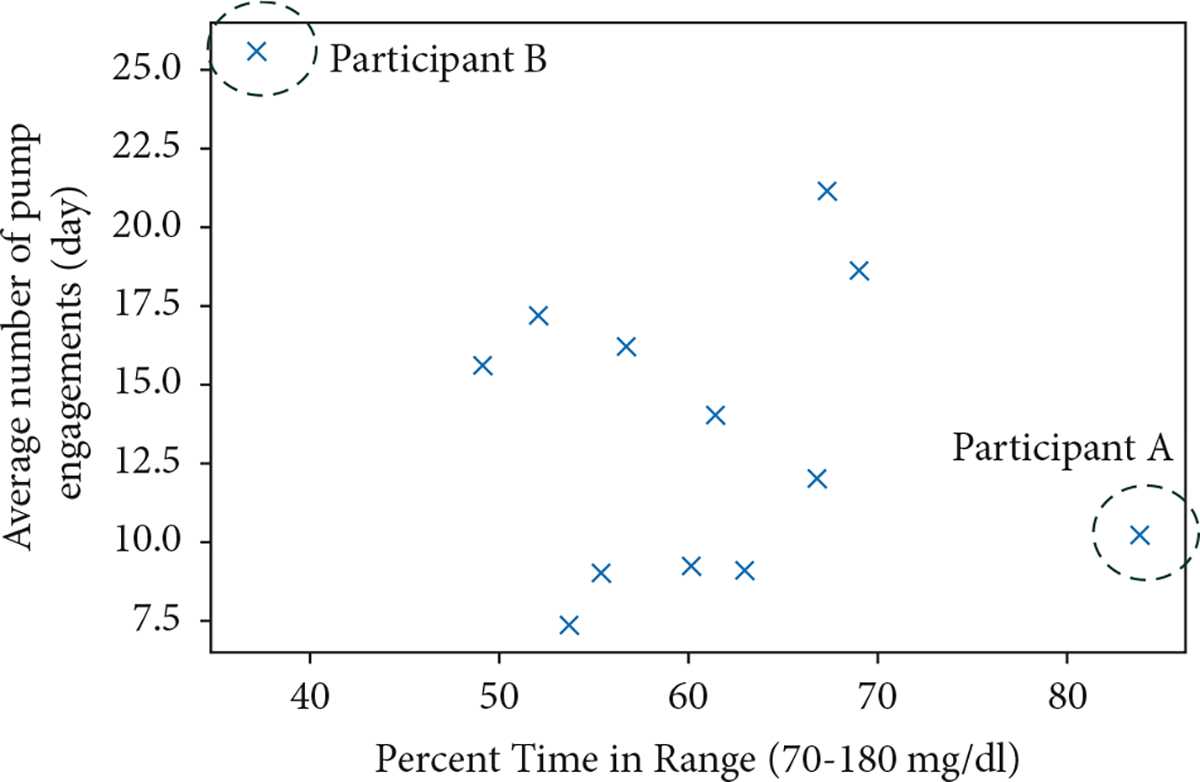
Between-person correlation between number of pump engagements per day and time in range 70–180 mg/dl (3.9–10.0 mmol/L).

There was no statistically significant correlation between the mean number of pump engagements and TIR (Figure 1). Two individuals were outliers in this distribution: “Participant A,” a 19 year-old female with T1D for 4.4 years, demonstrated an average of ~10 pump engagements/day and achieved an 85% mean TIR. Conversely, “Participant B” was a 16 year-old male who had diabetes for 1.9 years, averaged 26 pump engagements each day, and achieved a mean TIR of 36%.

When examining behavioral differences, Participant A bolused 5.4 times/day and entered an average of 198 grams of carbohydrate (CHO) into her bolus calculator each day (91% of all boluses). She acknowledged her CGM alerts 90% of the time. When asked to estimate her current glucose level, she was accurate 68% of the time. In contrast, Participant B bolused about the same number of times as Participant A (5 times/day), but rarely entered CHO into the bolus calculator (>1% of the time, averaging 1.6 grams of CHO per day), potentially making boluses less effective at controlling glucose levels. He further only acknowledged 28% of CGM alerts, possibly contributing to less awareness of his glucose levels, only estimating correctly 28% of the time.

### 3.2. Within-Person Predictors of Time in Range.

Next, patterns were analyzed within-person on a day-by-day basis. TIR was normally distributed across days (mean = 60.0% ± 17.5). Over the two-week period per participant, there was no significant change in the number of pump engagements/day (*p* = 0.13) or percent of CGM alerts acknowledged (*p* = 0.33); there was a nonsignificant trend toward improved TIR (*p* = 0.08), which we conservatively factored into our analyses.

In a univariate hierarchical regression, many situational awareness questions correlated to same-day TIR, as did several CGM engagement behaviors (Table 3). For situational awareness question #2 (“estimate your current glucose”) and question #4 (“predict your future glucose”), we ran separate analyses based on whether the participant had overestimated or underestimated their actual value; in both cases, the percentage of time that a participant made an underestimate was the better predictor of their same-day TIR. This pattern suggests that some types of perceptual errors may be more important than others to overall diabetes self-management. The total number of CGM alerts was negatively correlated with TIR, which is not surprising given that alerts signify out-of-range glucose value. However, the number of alerts acknowledged had a positive relationship with TIR, suggesting that attending to an alert may be an important component of diabetes self-management. Interacting with the pump more frequently and giving more boluses throughout the day were both associated with lower TIR. However, the percentage of boluses administered with carbohydrates entered was associated with better TIR.

Next, we ran multivariable models to test the underestimate of current glucose (situational awareness) alongside pump behaviors (Table 4). The number of CGM alerts, mean number of pump engagements/day, and percent of boluses that included a carbohydrate entry all remained significant for predicting both same-day and next-day TIR. The percent of CGM alerts acknowledged predicted same-day TIR. Importantly, the situational awareness variable—underestimating current glucose—continued to predict same-day TIR even after Control-IQ-relatedself-management behaviors were accounted for (*p* = 0.007). This finding suggests that situational awareness is a useful concept in understanding diabetes self-management beyond simply helping a person recognize when they need to deliver a bolus.

## 4. Discussion

This study is unique in examining daily patterns of situational awareness in addition to diabetes behaviors and glycemia in adolescents and young adults, a population that struggles with consistent diabetes care. This paper is the first to report that a person’s situational awareness of glucose levels predicts TIR on a daily basis, to some extent independent of diabetes self-management behaviors. We further demonstrated that not all pump engagements are effective for diabetes self-management, with a larger total number of Control-IQ interactions and boluses per day actually predicting lower same-day TIR results. On the other hand, interactions that involved acknowledging an alert message from the device or bolusing with a carbohydrate entry were both predictive of higher same-day TIR. Overall, these data provide new insights into the state of mind and behaviors of adolescent and young adult Control-IQ users that promote better glycemic control.

Our study found that a person’s situational awareness of their diabetes predicts better same-day TIR, and this was independent of when they last checked their glucose level (*p* = 0.8). This invites the question as to why some individuals can predict their glucose levels without referring to their device as recently, as if they are somehow more “in tune” with their diabetes, or “experts” in their own diabetes. Experts can notice “invisible” aspects of situations and respond to them based on a “feeling” or for reasons that they can’t articulate—features that are similar to the concept of situational awareness. Expertise is also suggested by the fact that TIR was better on days when participants more accurately predicted their future TIR as well. Underestimating one’s glucose level was predictive of lower TIR, perhaps because the person was less likely to be take measures to address hyperglycemia if they did not know they had high glucose levels. It is noteworthy that participants were asked to estimate their current glucose before looking at their device, which suggests a type of expertise.

Future interventions to promote diabetes self-management could encourage the development of situational awareness by helping new users to develop more expertise over time—for example, by training them to predict their own glucose in various scenarios and giving them feedback about the actual results. As adolescents gain more expertise monitoring their glucose levels and managing diabetes, these behaviors should become more automatic, and perhaps ultimately unnecessary as regular glucose-checking is replaced by a more intuitive form of expertise.

While hybrid closed loop systems may lower the burden of diabetes self-management compared to systems without automation, it is notable here that hyperglycemia is associated with more pump interactions, predicting a lower same-day TIR. The higher number of pump engagements are likely indicative of the user’s reactive attempts to monitor and mitigate hyperglycemia by checking glucose levels and giving correction boluses of insulin (which was also predictive of lower TIR in the same-day analysis). These data suggest that not all engagement is effective engagement; “reactive engagement” is less effective than “proactive engagement.” This is the first paper quantifying this negative effect of reactive engagement on glycemia. On the other hand, “proactive engagement,” such as programming boluses with carbohydrates, predicted a higher TIR compared to boluses that were given in response to hyperglycemia or programmed manually. It will be important in future studies to further tease apart the role of reactive and proactive engagement. Further, patient-reported outcome measures related to HCL currently focus on benefit of the system [19, 20] or overall diabetes distress [21, 22], but are not able to capture device/diabetes burden related to glycemic control.

The concept of “effective engagement” has been studied in digital interventions and should be considered in future research with how adolescents and young adults interact with HCL systems [23]. This study attempted to distinguish which type of Control-IQ system engagements are useful and effective, but future studies would benefit from working definitions of “effective engagements” in light of timing of engagement, reason for engagement, and accuracy of the response.

Strengths of this study include a sample of adolescents and young adults with a wide range of baseline glycemia and duration of diabetes, as well as novel measurements of situational awareness and diabetes behaviors spanning multiple days. We also report granular information about Control-IQ engagement behaviors, furthering the published literature on user behaviors [12]. The study’s major weakness was its small sample size, although we were able to partially compensate for this by using multilevel models to capitalize on the availability of multiple data points provided by each participant. Another weakness includes the fact that the majority of the samples were White non-Hispanic individuals, limiting the ability of these pilot findings to determine if there are racial/ethnic differences, and drawing attention to the importance of further testing in a more diverse sample. Although we asked participants to exclusively use the Control-IQ system to view glucose levels, it is possible that data were not captured if they chose to view glucose data on their phone application. Finally, situational awareness questions were only asked 2–3 times a day, which limited the number of data points and potentially led us to miss additional relationships with self-management.

## 5. Conclusion

Overall, the findings of this exploratory study should lead to future research on defining effective engagement behaviors and determining how to best design interventions to improve both situational awareness of diabetes and effective responses.

## Figures and Tables

**Table 1:** Participant characteristics

	Mean ± s.d	Range	Median (IQR)	*N (%)*
Age	18 ± 1.86	15–22	18.5 (2.8)	
Race/Ethnicity				12 (86%) White, non-Hispanic 1 (7%) Hispanic 1 (7%) Native Hawaiian or Pacific Islander
Duration of diabetes	8.0 ± 2.1	1.9–10.7	7.6 (2.3)	
Duration of control-IQ use				3 (21%) <1 year 8 (57%) 1-2 years 3 (21%) 2–3 years
Age at diagnosis	10.0 ± 2.2	3.8–14.1	9.5 (3.8)	
Last known HbA1c (%)	7.9% ± 1.2 (63 mmol/mol)	5.7–10.9 (39–96 mmol/mol)	7.7 (2.6) (61 mmol/mol)	
First parent education level				1 (7%) high school/GED 5 (36%) some college 6 (43%) bachelor degree 2 (14%) Postgraduate degrees
Second parent education level				1 (7%) no degree 4 (29%) high school/GED 1 (7%) some college 7 (50%) bachelor degree 1 (7%) postgraduate degree
Insurance status				10 (71%) private insurance 4 (29%) public insurance

**Table 2:** Situational awareness and pump behavior correlations with time in range (between-persons).

	*r*	*p* values

Mean % correct glucose estimate (Q2)	0.73	0.03
Mean % underestimate glucose (Q2)	−0.73	0.02
Mean % overestimate glucose (Q2)	0.47	0.25
Mean % correct direction glucose trending (Q3)	0.27	0.33
Mean % correct prediction 1 hour (Q4)	0.27	0.79
Mean % underestimate prediction (Q4)	−0.6	0.23
Mean % overestimate prediction (Q4)	0.57	0.27
Mean # CGM alerts/day	−0.43	0.01
% CGM alerts acknowledged	0.76	0.0001
Mean number of pump engagements/day	−0.36	0.86
Mean # boluses/day	0.33	0.13
Total grams of CHO entered into pump/day	0.18	0.07
Mean # boluses with carb entry/day	0.39	0.05

**Table 3:** Univariate predictors of daily time in range (within-person).

	*Prediction of same-day TIR*			*Prediction of next-day TIR*		
	Effect size (*r*)	*t*	*p*	Effect size (*r*)	*t*	*p*

% Correct glucose estimate (Q2)	+0.15	1.95	0.053	+0.13	1.56	0.12
% Underestimate glucose (Q2)	−0.31	−4.04	<0.001***	−0.15	−1.87	0.06
% Overestimate glucose (Q2)	+0.19	2.32	0.02*	+0.02	0.18	0.86
% Correct direction glucose trending (Q3)	+0.22	2.78	0.006**	−0.03	−0.36	0.72
% Correct prediction 1 hour (Q4)	+0.12	1.50	0.14	−0.01	−0.06	0.95
% Underestimate prediction (Q4)	−0.25	−3.16	0.002**	+0.06	0.75	0.45
% Overestimate prediction (Q4)	+0.18	2.28	0.02*	+0.08	−0.97	0.34
# CGM alerts received/day	−0.30	−3.38	0.001**	−0.15	−1.53	0.13
% CGM alerts acknowledged	+0.41	4.55	<0.001***	+0.28	2.85	0.005**
Mean # pump engagements/day	−0.28	−3.62	<0.001***	−0.02	−0.28	0.78
# Boluses given/day	−0.30	−3.90	<0.001***	+0.23	2.83	0.005**
Total grams of carbs entered/day	−0.02	−0.14	0.89	+0.10	0.85	0.40
Average grams of carbs per carb entry	+0.01	0.14	0.89	−0.11	−0.98	0.33
Total # of carb entries/day	+0.09	0.91	0.36	+0.20	2.05	0.04*
% Boluses that included carb entry	+0.41	4.63	<0.001***	+0.32	3.31	0.001**

* *p* < 0.05

** *p* < 0.01

*** *p* < 0.001.

**Table 4:** Final multivariate model predicting day-by-day time in range (70–180 mg/dl and 3.9–10.0 mmol/L) in adolescents and young adults with type 1 diabetes.

	*Prediction of same-day TIR*			*Prediction of next-day TIR*		
	Effect size (*r*)	*t*	*p*	Effect size (*r*)	*t*	*p*

% Underestimate glucose (Q2)	−0.22	−2.73	0.007**			
# CGM alerts received/day	−0.31	−3.43	<0.001***	−0.29	−2.71	0.008**
% CGM alerts acknowledged	+0.20	2.31	0.02*			
Mean # pump engagements/day	−0.27	−3.00	0.003**	−0.24	−2.59	0.011*
# Boluses given/day				+0.28	3.31	0.001**
% Boluses that included carb entry	+0.49	5.75	<0.001***	+0.49	4.89	<0.001***

* *p* < 0.05

** *p* < 0.01

*** *p* < 0.001.

## Data Availability

The data used in this study are available upon reasonable request.

## Abbreviations

CGM: Continuous glucose monitor
TIR: Time in range
